# The complete chloroplast genome sequence of the medicinal plant *Lepidium meyenii* Walp. (Cruciferae)

**DOI:** 10.1080/23802359.2020.1787256

**Published:** 2020-07-20

**Authors:** Feng-qin Zhang, Hai-zhu Zhang

**Affiliations:** a College of Pharmacy and Chemistry, Dali University, Dali, China; b Key Laboratory of Yunnan Provincial Higher Education Institutions for Development of Yunnan Daodi Medicinal Materials Resources, Yunnan, China

**Keywords:** *Lepidium meyenii* Walp, chloroplast, Illumina sequencing, phylogeny

## Abstract

*Lepidium meyenii* Walp. is a frequently used medicinal plant (namely, ‘maca’) in Yunnan Province of China. In this study, we sequenced the complete chloroplast (cp) genome sequence of *L. meyenii* to investigate its phylogenetic relationship in the family Cruciferae. The chloroplast genome of *L. meyenii* was 154,839 bp in length with 36.39% overall GC content, including a large single copy (LSC) region of 83,943 bp, a small single copy (SSC) region of 17,978 bp and a pair of inverted repeats (IRs) of 52,918 bp. The cp genome contained 103 genes, including 78 protein-coding genes, 21 tRNA genes, and four rRNA genes. The phylogenetic analysis indicated *L. meyenii* was closely related to the genus *Capsella*.

*Lepidium meyenii* is a 1-year or biennial herb of the *Lepidium* genus in the cruciferous family. It is native to the Andean region of South America with low temperatures, strong winds and harsh ecological conditions at an altitude of 3500 − 4500 m. Local people have a long history of planting and eating maca, and there are reports in the literature that they have been eating it for more than 5800 years (Li et al. 2018). There is no history of eating maca in our country, however, after the medicinal plant was included in the new food resource catalog in May 2011, the consumption of maca in China has a legal basis (Li et al. 2018). Lijiang and huize of yunnan are important producing areas for introducing *L. meyenii* in China. The main edible part of maca is its rhizome, also known as beetroot or Peruvian ginseng. The main edible part of *L. meyenii* is its rhizome, also known as beetroot or Peruvian ginseng, which has a long history as medicine and food, rich in nutrition and remarkable efficacy. Pharmacological activity studies show that *L*. *meyenii* has a variety of effects such as anti-fatigue, enhance immunity, improve fertility, anti-inflammatory, anti-cancer, antioxidant, anti-viral, lower blood pressure, relieve depression and treat women's menopause syndrome (Wang et al. 2007). However, until now, most of the studies for this species mainly focused on describing its chemical compositions (Zhou et al. 2016; Zhou 2017) with little involvement in its molecular biology. Here, we reported the complete chloroplast genome sequence of *L. meyenii* and revealed its phylogenetic relationships with other species in the Cruciferae.

Fresh and clean leaf materials of *L. meyenii* were sampled from Huize County, Yunnan, China (N26.41°, E103.27°); meanwhile, a voucher specimen (No. ZHZ001) was collected and deposited at the Herbarium of Medicinal Plants and Crude Drugs of the College of Pharmacy and Chemistry, Dali University. The total genomic DNA was extracted using the improved CTAB method (Doyle 1987; Yang et al. 2014), and sequenced with Illumina Hiseq 2500 (Novogene, Tianjing, China) platform with pair-end (2 × 300 bp) library. About 4.11 Gb of raw reads with 20,623,534 paired-end reads were obtained from high-throughput sequencing. The raw data was filtered using Trimmomatic v.0.32 with default settings (Bolger et al. 2014) were assembled into circular contigs using GetOrganelle.py (Jin et al. 2018). Finally, the cpDNA was annotated by the Dual Organellar Genome Annotator (DOGMA; http://dogma.ccbb.utexas.edu/) (Wyman et al. 2004) and tRNAscan-SE (Lowe and Chan 2016).

The annotated chloroplast genome was submitted to the GenBank with an accession number MT430983. The total length of the chloroplast genome was 154,839 bp, with 36.39% overall GC content. With typical quadripartite structure, a pair of IRs (inverted repeats) of 52,918 bp was separated by a small single copy (SSC) region of 17,978 bp and a large single copy (LSC) region of 83,943 bp. The cp genome contained 130 genes, including 78 protein-coding genes, 21 tRNA genes, and four rRNA genes. Of these, 17 genes were duplicated in the inverted repeat regions, 16 genes, and 6 tRNA genes contain one intron, while two genes (ycf3 and clpP) have two introns.

To investigate its taxonomic status, a total of 28 cp genome sequences of Cruciferae species were downloaded from the NCBI database used for phylogenetic analysis. After using MAFFT V.7.149 for aligning (Katoh and Standley 2013), a neighbor-joining (NJ) tree was constructed in MEGA v.7.0.26 (Kumar et al. 2016) with 1000 bootstrap replicates and two Cruciferae species (*Alyssum desertorum*:NC_034299 and *Alyssum gmelinii*: MF169880) were used as outgroups. The results showed that *L. meyenii* was closely related to the genus *Capsella* (Figure 1). Meanwhile, the phylogenetic relationship in Cruciferae was consistent with previous studies and this will be useful data for developing markers for further studies.

**Figure 1.**
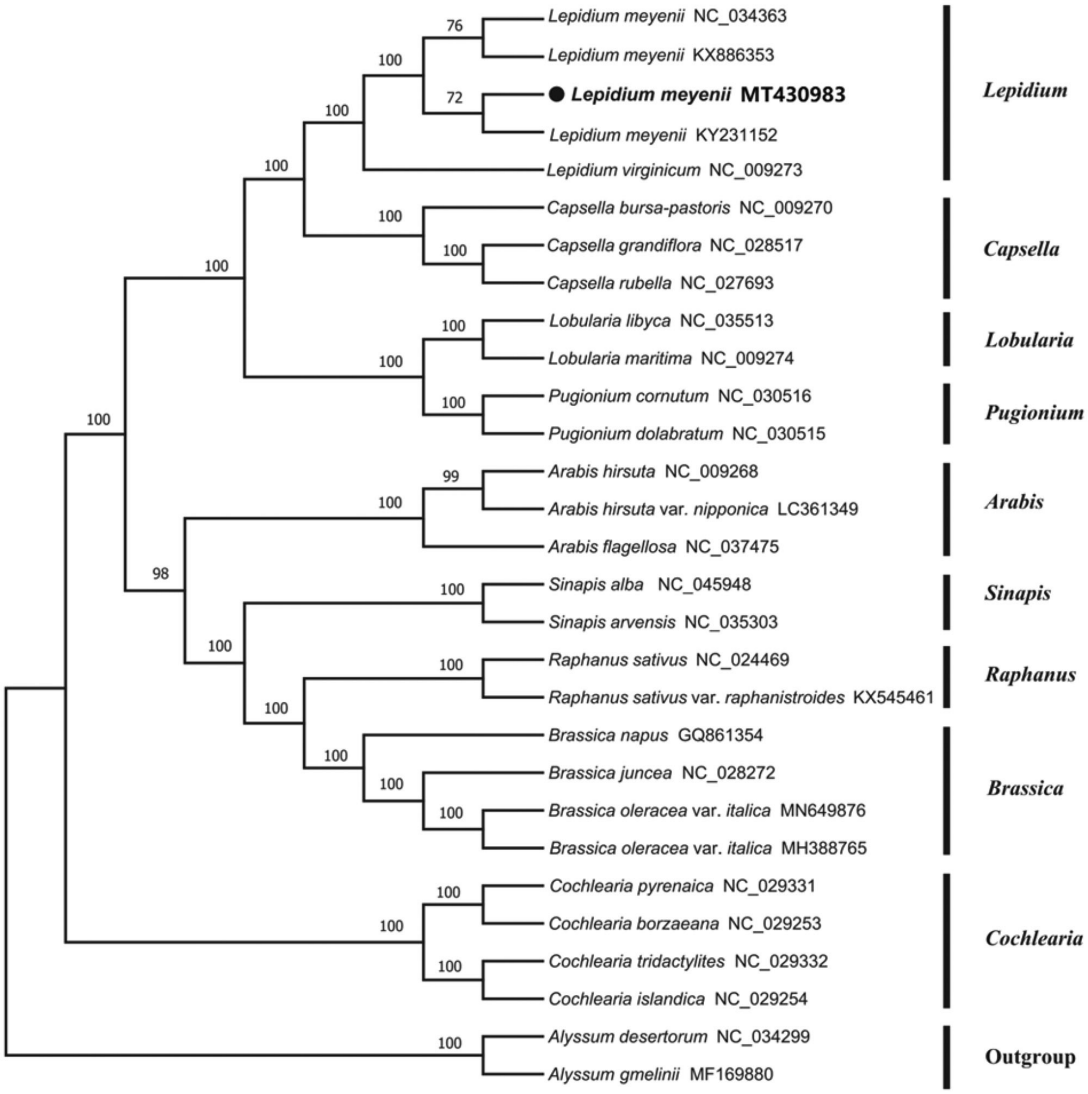
Neighbor-joining (NJ) tree of 29 species within the family Cruciferae based on the plastomes using two Cruciferae species as outgroups.

## Data Availability

Date available. Please access the GenBank and obtain it in https://www.ncbi.nlm.nih.gov/nuccore/MT430983.
